# A novel *BAG5* variant impairs the ER stress response pathway, causing dilated cardiomyopathy and arrhythmia

**DOI:** 10.1038/s41598-024-62764-y

**Published:** 2024-05-25

**Authors:** Rutairat Wongong, Anusak Kijtawornrat, Chalurmpon Srichomthong, Siraprapa Tongkobpeth, Phichittra Od-Ek, Adjima Assawapitaksakul, Natarin Caengprasath, Apichai Khongphatthanayothin, Thantrira Porntaveetus, Vorasuk Shotelersuk

**Affiliations:** 1 Center of Excellence for Medical Genomics, Department of Pediatrics, Faculty of Medicine, Chulalongkorn University, Bangkok, Thailand; 2 Excellence Center for Genomics and Precision Medicine, King Chulalongkorn Memorial Hospital, the Thai Red Cross Society, Bangkok, Thailand; 3 Department of Physiology, Faculty of Veterinary Science, Chulalongkorn University, Bangkok, Thailand; 4 Center of Excellence in Arrhythmia Research Chulalongkorn University, Department of Medicine, Faculty of Medicine, Chulalongkorn University, Bangkok, Thailand; 5 Center of Excellence in Genomics and Precision Dentistry, Department of Physiology, Faculty of Dentistry, Chulalongkorn University, Bangkok, 10330 Thailand; 6 Graduate Program in Geriatric and Special Patients Care, Faculty of Dentistry, Chulalongkorn University, Bangkok, Thailand; 7 Bangkok Heart Hospital, Bangkok, Thailand

**Keywords:** BAG5, Dilated cardiomyopathy, Cardiac arrhythmia, Endoplasmic reticulum stress, Tunicamycin, Unfolded protein response, Genetics, Medical genetics, Genetics research, Cardiovascular biology

## Abstract

Pathogenic *BAG5* variants recently linked to dilated cardiomyopathy (DCM) prompt further investigation into phenotypic, mutational, and pathomechanistic aspects. We explored the clinical and molecular characteristics of DCM associated with *BAG5* variants, uncovering the consistently severe manifestations of the disease and its impact on the endoplasmic reticulum (ER) stress response. The analysis involved three siblings affected by DCM and arrhythmia, along with their four unaffected siblings, their unaffected father, and their mother who exhibited arrhythmia. The parents were consanguineous. Exome and Sanger sequencing identified a novel *BAG5* variant, c.444_445delGA (p.Lys149AsnfsTer6), homozygous in affected siblings and heterozygous in parents and unaffected siblings. We generated heterozygous and homozygous *Bag5* point mutant knock-in (KI) mice and evaluated cardiac pathophysiology under stress conditions, including tunicamycin (TN) administration. *Bag5−/−* mice displayed no abnormalities up to 12 months old and showed no anomalies during an exercise stress test. However, following TN injection, *Bag5−/−* mice exhibited significantly reduced left ventricular fractional shortening (LVFS) and ejection fraction (LVEF). Their cardiac tissues exhibited a notable increase in apoptotic cells, despite non-distinctive changes in CHOP and GRP78 levels. Interestingly, only *Bag5* KI male mice demonstrated arrhythmia, which was more pronounced in *Bag5−/−* than in *Bag5*+/−males. Here, our study reveals a novel *BAG5* mutation causing DCM by impairing the ER stress response, with observed sex-specific arrhythmia differences.

## Introduction

Dilated cardiomyopathy (DCM), characterized by a progressive course of ventricular dilation and systolic dysfunction, is a severe heart disease that can lead to heart failure and sudden cardiac death (SCD). The prevalence of DCM is reported as 1 in 250 individuals^1^. The etiology includes genetic mutations in at least 60 distinct genes^2^.

Recent novel findings have unveiled that variants in the Bcl-2 associated athanogene 5 (*BAG5*) gene are responsible for DCM in four unrelated families^3^. *BAG5*¸ a member of the BAG family of molecular chaperone activity regulators, encodes a protein that belongs to the BAG1-related proteins family, known for its pivotal role in cellular and cardiac function^4^. These proteins exhibit differential expression patterns in cases of heart failure^5^ and are found to be up-regulated under stressful conditions^6^. *BAG5* has previously been found to protect cardiomyocytes from endoplasmic reticulum (ER) stress-mediated apoptosis and improves cellular viability.^4^

In 2022, Hakui et al*.* identified three de novo homozygous truncating variants in *BAG5* among patients with inherited DCM. These patients presented with severe left ventricular dysfunction, early-onset heart failure symptoms, and a high incidence of refractory ventricular arrhythmias. Notably, all individuals carrying homozygous or compound heterozygous *BAG5* variants developed DCM, highlighting a strong genotype–phenotype correlation. In contrast, heterozygous carriers remained unaffected^3,7^. Subsequently, Inoue et al*.* reported two unrelated cases of DCM caused by compound heterozygous truncating variants in *BAG5*. Both patients presented with advanced heart failure and experienced severe ventricular arrhythmias. Immunohistochemical analysis of heart tissue from these patients revealed a significant reduction in BAG5 and SERCA2 protein expression. These findings further support the role of biallelic *BAG5* variants in causing DCM^8^.

Despite these significant studies, the current understanding of the phenotypic and genotypic spectra of *BAG5*-associated DCM and its pathomechanism remains incomplete. In this study, we studied a large family with three siblings affected with *BAG5*-associated DCM. Homozygous *Bag5* point mutant knock-in (KI) mice were generated to investigate DCM pathophysiology.

## Results

### Phenotypic features of patients with DCM

**Figure 1:**
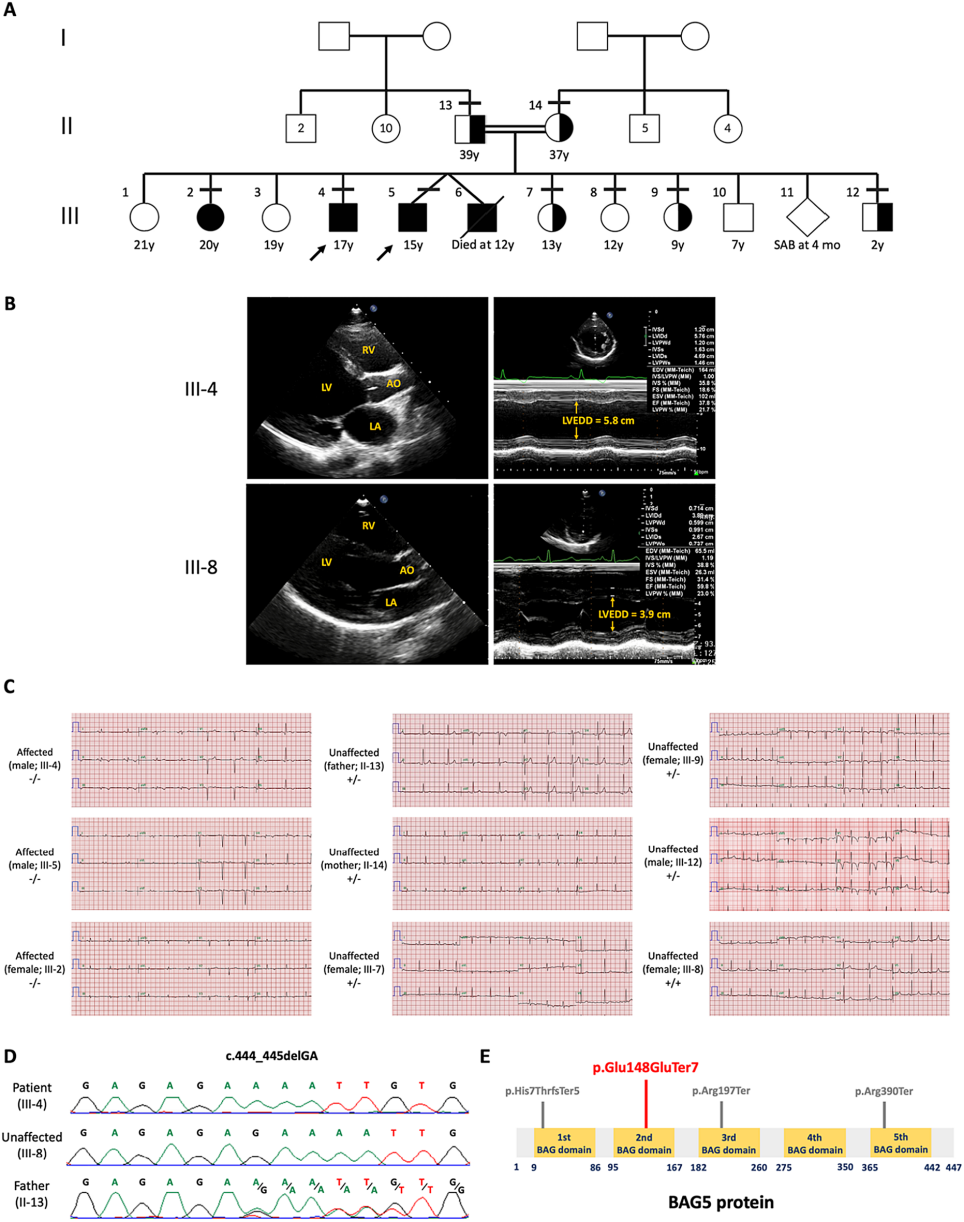
Family pedigree, cardiac features and genotype of the patients. (**A**) The family pedigree showing four patients (blackened symbols) with one deceased (diagonally crossed). Horizontal lines above the individual’s symbols denote the available detailed clinical information and DNA for genetic studies. The number inside individual’s symbols represents multiple members. (**B**) 2D (upper left) and M-mode (upper right) echocardiographic images of the proband (III-4) showing dilated left ventricle (LV) with end-diastolic dimension (EDD) of 5.8 cm, Z score =  + 4.8 SD and poor LV ejection fraction (LVEF, 38%) compared to unaffected family member (III-8) having normal LVEDD (3.9 cm, Z score =  + 0.2SD) and normal LVEF (60%). RV = right ventricle, LV = left ventricle, AO = ascending aorta, LA = left atrium, LVEDD = left ventricle end-diastolic dimension. (**C**) ECG assessments showed that all affected (III-2, III-4, and III-5) had a low voltage of the limb lead, T wave inversion in the infero-lateral leads and poor R progression in precordial leads. The father’s ECG (II-13) was normal. The mother’s ECG (II-14) exhibited intermediate abnormalities (borderline low voltage in limb leads, flat T wave in all the leads, and borderline poor R progression in precordial leads). The ECG of two out of three siblings with heterozygous variant carrier were normal (III-7, III-12) while the other female carrier showed borderline or mild abnormality (T wave inversion from V1-V4, III-9). III-8 who was the only available family member with a normal genotype showed normal ECG. (**D**) Sanger sequencing showing the homozygous *BAG5,* c.444_445delGA (p.Lys149AsnfsTer6) in the proband and wild-type in III-8, and heterozygous in the father. (**E**) Schematic diagram of BAG5 protein demonstrating *BAG5* mutations that have been reported. Red line indicates the variant identified in our patients affected with DCM (p.Lys149AsnfsTer6), while grey lines represent the variants identified by Hakui et al*.,* (2022).

A family of Middle Eastern ethnic with four siblings with DCM (III-2, III-4, III-5 and III-6) was studied. They were born to healthy consanguineous parents, although the specific degree of consanguinity within the extended family could not be determined (Fig. 1A). Probands were two brothers, III-4 and III-5, aged 17 and 15 years, whose cardiological evaluations showed definite DCM with left ventricular ejection fraction (LVEF) by echocardiography of 40% and 36%, respectively (Fig. 1B). III-2 had normal LV size (end-diastolic volume 90.4 mL) with mild LV dysfunction (LVEF 46%) and mild to moderate LV global hypokinesia on cardiac MRI. III-6 passed away at the age of 12 years old while waiting for a heart transplantation. The echocardiogram of the parents (II-13 and II-14) and other siblings (III-7, III-8, III-9, III-12) showed neither DCM nor non-compaction (Table S1).

Electrocardiogram (ECG) assessments showed that all available patients, III-2, III-4, and III-5 had a low voltage of the limb lead, T wave inversion in the infero-lateral leads, and poor R progression in precordial leads. Their 39-year-old father’s ECG was normal while the 37-year-old mother’s ECG exhibited intermediate abnormalities (borderline low voltage in limb leads, flat T wave in all the leads, and borderline poor R progression in precordial leads). The ECG for the three heterozygous carriers showed that III-7, III-12 had normal findings while III-9 showed borderline or mild abnormality (T wave inversion from V1-V4). III-8 who harbored a normal *BAG5* genotype showed normal ECG (Fig. 1C, Table S1).

### Identification of biallelic frameshift variant in *BAG5* in patients with DCM

Exome sequencing identified that all available three patients, III-2, III-4, and III-5 were homozygous, while the parents were heterozygous for the c.444_445delGA (p.Lys149AsnfsTer6) in *BAG5* (ClinVar accession number: VCV003918808.1; submitted by our group). Subsequent Sanger sequencing of additional family members showed that the c.444_445delGA variant was segregated with the phenotypes across nine available members of the family. II-13, II-14, III-7, III-9, III-12 were heterozygous for the mutant allele and III-8 was homozygous for the wild-type allele (Fig. 1D). This variant is the fourth identified pathogenic mutation in *BAG5* causing DCM (Fig. 1E).

## Absence of cardiac abnormalities in the KI mice without intervention and during an exercise stress test

To investigate the role of BAG5 in cardiac functions, we generated heterozygous and homozygous *Bag5* point mutant KI mice. Without intervention, all mice were healthy up to 3 months of age. To comprehensively evaluate the potential progression of cardiac abnormalities, we extended our examinations to older mice at 6 and 9 months of age. Surprisingly, echocardiography results obtained from KI 6 and 9 months of age also did not reveal any significant differences when compared to wild-type (WT) mice (Supplementary Fig. S3).

We consequently conducted the graded maximal exercise test (GXT) of *Bag5*+/+, +/−, −/− mice within the age range of 12–22 months to evaluate cardiovascular fitness (CVF) during prolonged exercise session, but no significant differences between groups were found (Table S2).

### Compromised cardiac functions in *Bag5−/−* mice following the induction of ER stress

**Figure 2:**
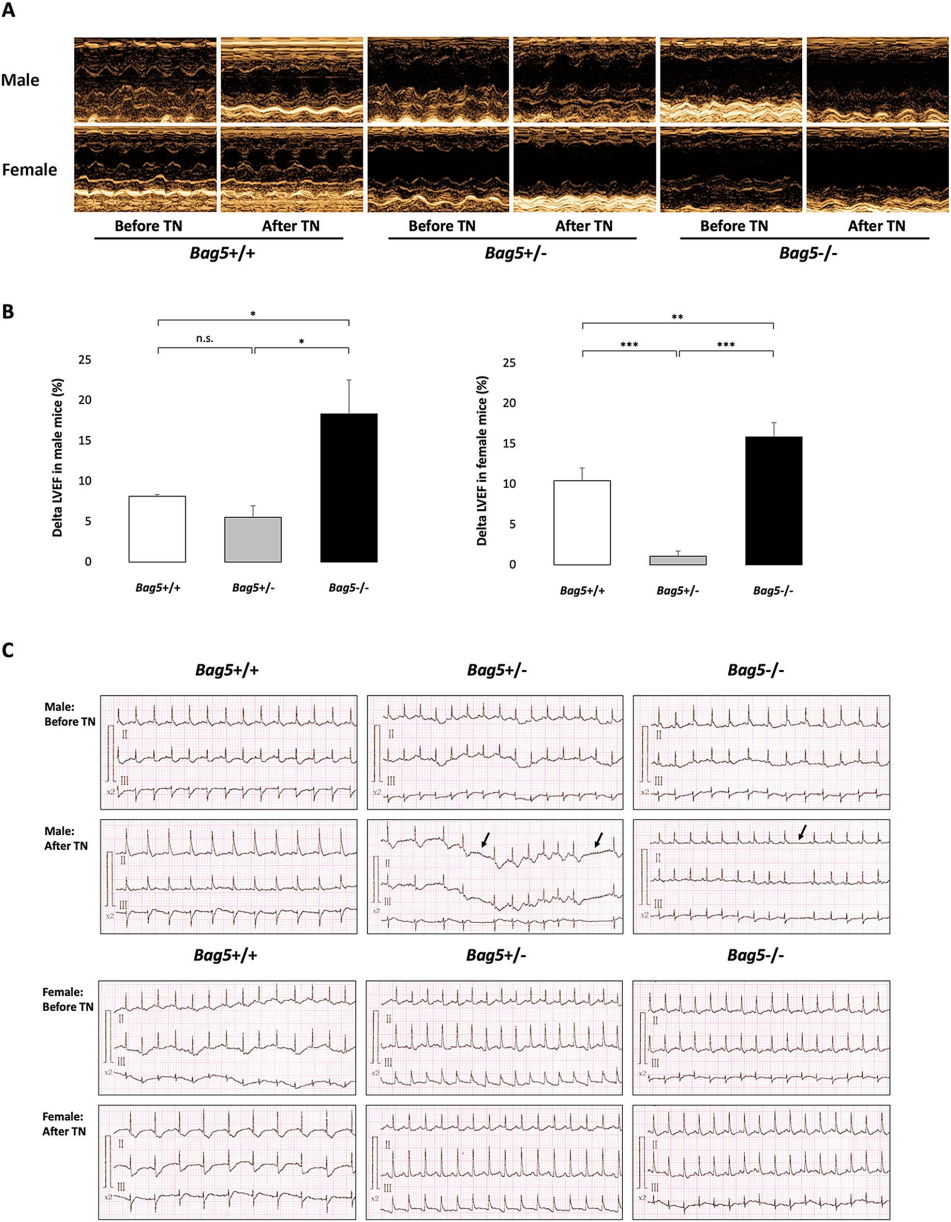
Cardiac functions in male and female *Bag5*-knock-in mice after the induction of ER stress. (**A**) M-mode echocardiography images in male (upper) and female (lower) of *Bag5*+/+, *Bag5*+/−, and *Bag5−/−* mice. (**B**) Changes in left ventricular ejection fraction (LVEF) in male (left) and female (right) of *Bag5*+/+, *Bag5*+/−, and *Bag5−/−* mice. Results are shown as the mean ± standard deviation (n = 3). n.s., not significant, * *P* < 0.05, ** *P* < 0.01, and *** *P* < 0.001. (**C**) Representative electrocardiographic recordings (Lead I, II, and III) before and after intraperitoneal injection of TN (2 mg/kg) in both male and female *Bag5*+/+, *Bag5*+/−, and *Bag5−/−* mice. Black arrow indicates sinus pause. The ECG paper speed is 50 mm/s and the calibration is 20 mm/mV.

ER stress has emerged as a significant contributor to the pathogenesis of heart disease.^9^ In this study, we employed TN injected intraperitoneally as an ER stress inducer, based on a previous report by Gupta et al. (2016) demonstrating the possible involvement of BAG5 in ER stress response and apoptosis in cardiomyocytes^4^. Following TN administration, *Bag5*−/− mice showed the most enlargement of EDV and ESV compared with *Bag5*+/− and *Bag5*+*/*+ mice (Fig. 2A, Table S3-S4). Echocardiographic assessments revealed a significant increase in delta LVEF in *Bag5*−/− mice compared to their pre-injection levels and compared with *Bag5*+/− *and Bag5*+*/*+ mice (Fig. 2B). *Bag5*−/− mice showed the most significant reductions in LVEF and LVFS compared with *Bag5*+/− and *Bag5*+*/*+ mice after receiving TN (Table S3–S4). Notably, no discernible histological abnormalities were evident (Supplementary Fig. S4). Unfortunately, one male *Bag5*−/− mouse died suddenly within 72 h after TN injection without obvious explanation.

Furthermore*,* ECG showed that one *Bag5*−/− and one *Bag5*+/− males, but not females, experienced arrhythmia after TN injection which indicated by long electrical standstills (sinus pause) (Fig. 2C). Overall, of the three *Bag5−/−* males, one died suddenly, and the second one had arrhythmia.

### ER stress regulation and apoptosis affected by the loss of *Bag5*

**Figure 3:**
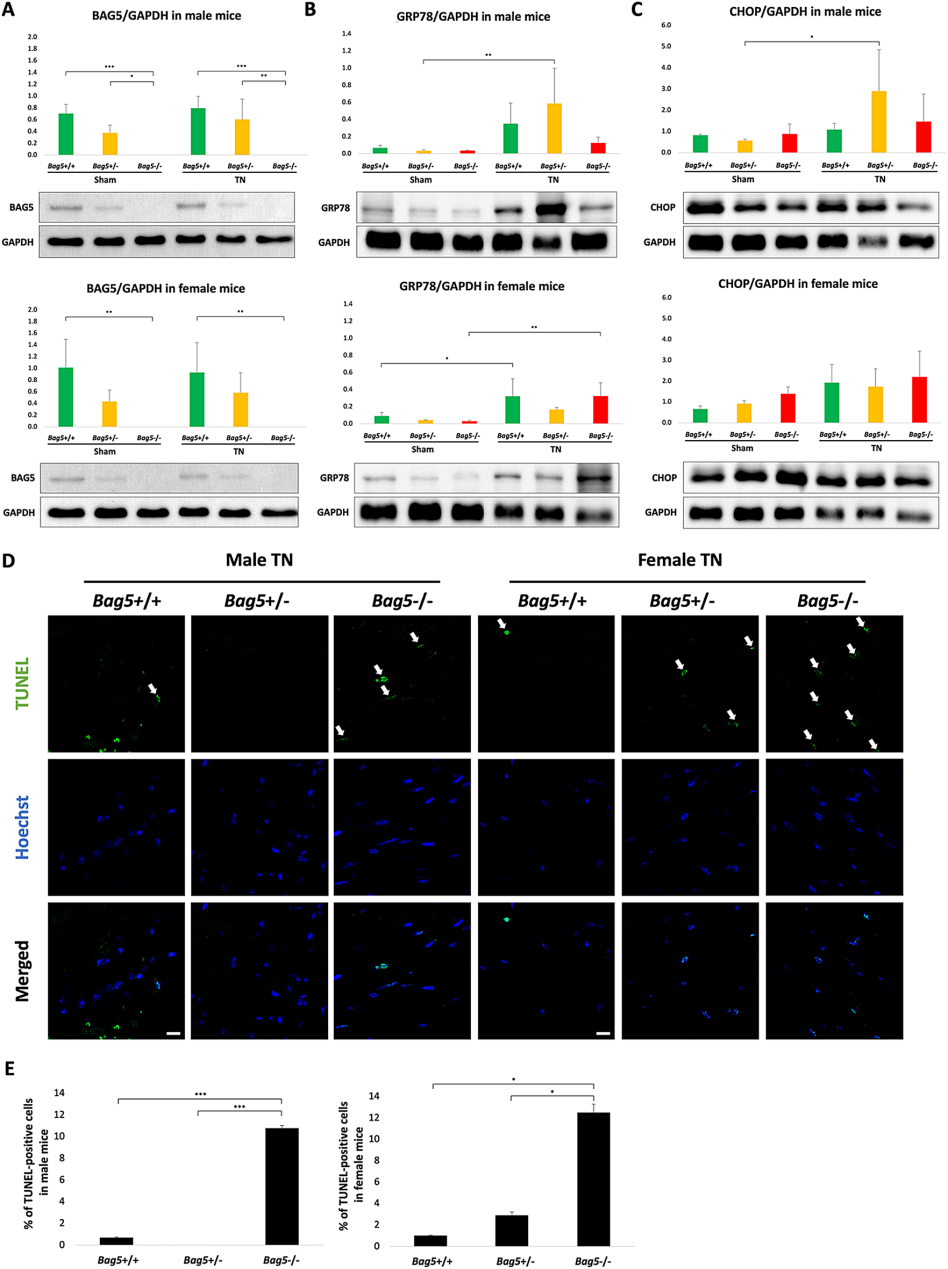
Studies of GRP78, CHOP and apoptosis in *Bag5*-knock-in mice after the induction of ER stress. (**A**) Western blot showing the absence of Bag5 protein in male (upper panel) and female (lower panel) *Bag5−/−* mice. (**B**) GRP78 expression in male (upper panel) and female (lower panel) of *Bag5*+/+, *Bag5*+/−, and *Bag5−/−* mice. (**C**) CHOP expression in male (upper) and female (lower) of *Bag5*+/+, *Bag5*+/−, and *Bag5−/−* mice. Data are presented as mean ± standard deviation (SD) of triplicate analyses from a single sample per genotype (n = 1). (**D**) Representative confocal images of fixed cardiac tissue from 12-week-old *Bag5*+/+, *Bag5*+/−, and *Bag5−/−* mice administered TN. TUNEL positive cells (arrows) were labeled with green fluorescence and cell nuclei were labeled with Hoechst (blue), scale bar 10 μm. (**E**) Percentage of TUNEL positive cells in male (left) and female (right) of *Bag5*+/+, *Bag5*+/−, and *Bag5−/−* mice after TN injection. Data are presented as mean ± SD of 10 fields per heart sample from a single sample per genotype (n = 1). *P < 0.05, **P < 0.01, and ***P < 0.001.

Considering the potential involvement of ER stress in DCM among *Bag5* point mutant KI mice, we further examined the ER stress signaling pathway and the participation of glucose-regulated protein (GRP78) and C/EBP Homologous Protein (CHOP) in response to TN, both of which are implicated in the unfolded protein response (UPR) and ER stress-induced apoptosis. Western blot analysis revealed that the expression of Bag5 protein in cardiac tissue was completely absent in *Bag5−/−* mice and decreased in *Bag5*+/− mice, when compared to that in *Bag5*+*/*+ mice (Fig. 3A, Supplementary Fig. S5). All mice treated with TN exhibited elevated expression of GRP78 and CHOP. Notably, the observed changes did not display distinguishable trends among *Bag5−/−, Bag5*+/−*,* and *Bag5*+*/*+ mice (Fig. 3B,C, Supplementary Fig. S5). To further assess apoptosis, TUNEL assay of the TN-treated cardiac tissues showed that there was a significant increase in apoptotic cell death in *Bag5−/−* mice, compared to *Bag5*+*/*+ and *Bag5*+/− mice (Fig. 3D,E). These results demonstrate a hyper-activation of ER-stress apoptosis in Bag5 deficiency related DCM.

## Discussion

DCM represents the most prevalent form of cardiomyopathy and exhibits a rapidly expanding genetic basis. In 2022, Hakui et al*.* identified three de novo homozygous variants in *BAG5* including c.589C > T (p.Arg197Ter), c.1168C > T (p.Arg390Ter) and c.18dupA (p.His7ThrTer5) in the patients with inherited DCM^3^. Inoue et al. (2023) further reported two compound heterozygous truncating variants in the *BAG5* gene, c.1168C > T (p.Arg390Ter) and c.589C > T (p.Arg197Ter), in individuals presenting with advanced heart failure attributed to DCM^8^. However, DCM's genetic underpinnings are notably diverse, necessitating comprehensive information to delineate the phenotypic and mutational spectra associated with *BAG5*-related DCM, as well as to elucidate its underlying pathomechanism.

In this study, a novel homozygous 2-bp deletion variant c.444_445delGA (p.Lys149AsnfsTer6) in *BAG5* associated with familial DCM adds to the growing list of pathogenic variants within *BAG5* gene associated with DCM, underscoring the genetic heterogeneity of this condition, and suggesting that these *BAG5* mutations are loss-of-function variants.

The individuals diagnosed with DCM in our study originated from a large consanguineous family. ECG findings indicated low limb lead voltage, T-wave inversion in infero-lateral leads, and poor R-wave progression in precordial leads in all available patients (III-2, III-4, and III-5). Their mother (II-14) displayed intermediate ECG abnormalities, including borderline low voltage in limb leads, flat T-waves in all leads, and borderline poor R-wave progression in precordial leads. Additionally, one female sibling (III-9) exhibited mild or borderline abnormality with T-wave inversion from V1 to V4, while the 39-year-old father did not show abnormalities in ECG, suggesting a potential predisposition to arrhythmia in female carriers of the *BAG5* variant.

In this study, the clinical features of patients with *BAG5*-related DCM, including severe cardiac dysfunction, early-onset disease, and abnormal ECG results. Particularly notable was the consistent alignment of early-onset DCM presentation caused by biallelic truncating variant in the *BAG5* gene, mirroring findings from previous studies^3,8^. This alignment with existing research highlights the unique nature of *BAG5*-related DCM compared to other genetic forms of the condition. Unlike the broader spectrum seen in *BAG3*-related DCM, characterized by varying severity of heart failure and age of onset^10^. Our study suggests a connection between *BAG5* gene variants and clinical presentation. This clarification emphasizes the significance of our research, highlighting the unique clinical features and genetic associations specific to *BAG5*-related DCM.

We found that cardiac arrhythmia is a sex-specific, found only in male KI mice, irrespective of monoallelic or biallelic *BAG5* mutations. Of the three TN-treated *Bag5*+/− male mice, one (33%) had arrhythmias. Of the three TN-treated *Bag5−/−* male mice, one (33%) had arrhythmias and another one died suddenly without obvious explanation. In numerous cases, arrhythmias are a major contributor to sudden cardiac death, a common and life-threatening complication in DCM patients^11,12^. None of the *Bag5−/−* and *Bag5*+/− female mice manifested arrhythmia.

In our study, among human carriers (genotype+/−), abnormal ECG activity was observed exclusively in females (2/3), with no such abnormalities detected in males (0/2). Conversely, within the mutant mouse model, abnormal ECG patterns were exclusively detected in males, affecting both heterozygous (1/3) and homozygous (1/3), (Table S5). We postulate that these sex differences may be attributed to variances in fundamental physiology, hormones, and differences in immune and fibrotic responses to cardiac tissue damage. While our study indicates a potential gender difference in the phenotype of *Bag5* KI mice, the underlying reasons remain elusive, mirroring the ambiguity observed in human *BAG5*-related DCM. Additionally, recent reports have highlighted infertility in *Bag5* knockout mice, further emphasizing the importance of BAG5 in reproductive physiology^13^. Notably, the enrichment of BAG5 expression in the testes, according to The Human Protein Atlas (https://www.proteinatlas.org/), underscores its potential role in male reproductive function. Nevertheless, to substantiate these findings, further investigation with larger sample sizes is warranted.

In the absence of intervention, *Bag5−/−* and *Bag5*+/− mice exhibited comparable LVEF values to age-matched *Bag5*+*/*+ mice, with all groups maintaining LVEF levels above 60%. A prior study demonstrated that the GXT reliably delivered data pertaining to the cardiovascular fitness (CVF)^14^. In order to evaluate the CVF over extended exercise durations, a GXT was conducted. Surprisingly, this evaluation did not uncover any noteworthy differences in the *Bag5* KI mice when compared to the WT mice. These unexpected outcomes challenged our initial hypothesis that the KI mice would exhibit notable cardiac abnormalities, prompting us to explore whether DCM phenotypes in these *Bag5* point mutant KI mice might be manifest only under certain conditions, such as ER stress stimulation.

To develop DCM in *Bag5* point mutant KI mice, ER stress is required. After using TN to induce ER stress, we observed a significant reduction in LVEF and LVFS in *Bag5−/−* mice treated with TN. The role of ER stress has emerged as a significant player in the pathogenesis of heart disease^9,15,16^. In response to an array of stressors such as oxidative stress, hypoxia, or accumulation of misfolded proteins, the UPR is activated, a complex signaling pathway initiated by the ER. The TUNEL assay revealed a greater number of apoptotic cells in the hearts of *Bag5−/−* mice in comparison to those in *Bag5*+/− and *Bag5*+*/*+ mice. These findings suggest that BAG5 might play a protective role in the ER stress response and cardiomyocyte apoptosis.

We further explored whether the underlying mechanism involved GRP78 and CHOP. The chaperon heat shock protein GRP78, also known as BiP (binding immunoglobulin protein) and the HSPA5 (heat shock protein family A (Hsp70) member 5) serve as pivotal regulators of the UPR. In patients with heart failure, there is an observed elevation in *GRP78* mRNA levels, indicating an activation of UPR and concurrent ER stress in this pathological state^16^. GRP78 works in conjunction with BAG5 to prevent the accumulation of misfolded proteins in the ER, while also modulating downstream signaling cascade that instigates the UPR. This leads to the phosphorylation of protein kinase RNA-activated-like ER kinase (PERK) and eukaryotic initiation factor 2α-subunit (eIF2α), subsequently leading to a reduction in CHOP protein levels, ultimately alleviating ER stress in cardiomyocytes^4^. CHOP is a transcriptional factor that mediates ER stress–induced apoptotic cell death^17^. Our observations revealed an escalating trend in GRP78 and CHOP proteins within the cardiac tissue after treated with TN, indicating prolonged ER stress potentially leading to cell death.

Previous research has demonstrated that CHOP’s pivotal role in dilated cardiomyopathy stemming from abnormal protein folding and ER stress^15^. CHOP-deficient mice displayed reduced cardiac hypertrophy, fibrosis, apoptotic cell death, and cardiac dysfunction relative to WT mice after transverse aortic constriction^16^. However, although expressions of GRP78 and CHOP were found to be elevated in all mice treated with TN, the observed changes did not significantly differ among *Bag5−/−, Bag5*+/−*,* and *Bag5*+*/*+ mice (Fig. 3B,C, Supplementary Fig. S5). These findings suggest that BAG5 might potentially exert a protective role in mitigating ER stress-induced cardiomyocyte apoptosis through alternative pathways, possibly involving ATF6 or XBP1, warranting further exploration.

In addition to ER stress, other forms of proteotoxic stress such as mitochondrial stress, oxidative stress, the ubiquitin–proteasome system (UPS), the autophagy-lysosome pathway, and stress affecting organelles like the Golgi apparatus and lysosomes could potentially contribute to the pathobiology of BAG5-related cardiomyopathy. For instance, oxidative stress and mitochondrial dysfunction contribute to heart disease by promoting cardiomyocyte dysfunction, inflammation, and fibrosis. Excessive production of reactive oxygen species (ROS) from dysfunctional mitochondria damages cellular components and triggers apoptotic cell death pathways, leading to myocardial remodeling and impaired cardiac function^18^. A limitation of our study is that we did not extensively explore these alternative proteotoxic stress pathways. Future studies that aim to broaden our understanding of proteotoxic stress beyond ER stress will allow us to gain a more holistic view of the pathobiology underlying BAG5-related cardiomyopathy.

In summary, our research unveils a novel homozygous 2-bp deletion c.444_445delGA, p.(Lys149AsnfsTer6) in *BAG5*, leading to DCM. Notably, we observe a heightened susceptibility to arrhythmias among female carriers in humans, as well as in heterozygous and homozygous *Bag5* mutant male mice. Moreover, our results propose a pathomechanistic cascade involving ER stress and apoptosis in *BAG5*-related DCM.

## Methods

### Ethics declarations

Human subject research was approved by the Institutional Review Board, Faculty of Medicine, Chulalongkorn University (IRB No. 264/62, Date of approval: 18^th^ July 2019), and in accordance with the Declaration of Helsinki of 1964 and its later amendments. Written informed consent was obtained from all participants for both participation and publication purposes. Animal research was approved by the Institutional Animal Care and Use Committee (IACUC) of Chulalongkorn University Laboratory Animal Center (Approval no. 2173007, Approval Date 9^th^ June 2021). The study was complied with the National Institutes of Health (NIH) Guide for the Care and Use of Laboratory Animals and the ARRIVE guidelines.

### Exome sequencing (ES) and sanger sequencing

Genomic DNA was extracted from peripheral blood leukocytes of nine members of a family, comprising three affected and four unaffected siblings and their consanguineous parents (Fig. 1A). Genetic analysis was conducted according to a previous study^19^. ES was performed for two affected siblings (III-2 and III-4), and their parents using the Illumina HiSeq 4000 instrument (Illumina, SD, USA). Genetic analysis was performed according to a previous study^20^. Briefly, single nucleotide variants (SNVs) and Indels were filtered by the following filtering criteria; (1) consistent with autosomal recessive mode of inheritance, (2) not synonymous, (3) less than 1% in the 1000 Genomes Project and Genome Aggregation Database (gnomAD: gnomad.broadinstitute.org), and (4) located in exons or flanking introns of genes related to cardiomyopathy (HP: 0001638) and in genes linked to DCM according to previous studies^2,21^. The identified c.444_445delGA variant in *BAG5* (NM_001015049.2) was screened by Sanger sequencing in other family members, using the primers: 5′-GGTGCCTCTGAAGACAGAAAGGGAATC-3' and 5’-AAGCTGGGGCGGAGCTGGAGA-3'. The exome data had been submitted to the GenBank database (Submission number: SUB14390967).

### Generation of the *Bag5* mutant knock-in (KI) mice

The homozygous *Bag5* point mutant knock-in (KI) mice were generated by Genoway (Lyon, France) from the C57BL/6N background. Briefly, a targeting strategy was devised to create a constitutive mutant model, involving the deletion of two nucleotides (delCA), positioned 346-bp downstream of the 5' end of exon 3 that corresponded to the human delGA mutant (Supplementary Fig. S1). The genomic region containing the targeted *Bag5* mouse gene was isolated from the C57BL/6N mouse genomic DNA which was isogenic with the embryonic stem cell line employed for homologous recombination (HR). A second vector was created to replicate the DNA conformation at the targeted locus post HR, linking the short homology arm of the targeting vector with the *Bag5* locus. This positive control vector functioned as a check for the PCR primers utilized in the screening tests. The DNA sequence analysis was conducted to confirm the accuracy of the final targeting vector. Subsequently, the recombined embryonic stem cell clones obtained were injected into blastocysts, which were then implanted into pseudo-pregnant female mice. These mice were allowed to develop to term. A minimum of two chimeras were generated. Once the chimeras reached a suitable age, they were mated with Cre-deleter females to achieve germline transmission and excise the Neo-cassette, ultimately resulting in the production of the heterozygous constitutive point mutant mice. All mice were housed in the specific pathogen-free (SPF) facilities at the Chulalongkorn University Laboratory Animal Center (CULAC), Bangkok, Thailand maintained at 21 °C and 50 ± 10% relative humidity under a 12 h/ 12 h light/dark cycle (6:00 am–6:00 pm).

### Genotyping of the mice

The polymerase chain reaction (PCR) genotyping was performed on the offspring at three weeks of age to differentiate *Bag5*+*/*+, *Bag5*+/−, and *Bag5−/−* mice (Supplementary Fig. S2). Ear or tail tissues were extracted and amplified using KAPA Mouse Genotyping Kit (KAPA Biosystems, #KK7302) with primers: 5’-GGTGCCTCTGAAGACAGAAAGGGAATC-3’ and 5’-AAGCTGGGGCGGAGCTGGAGA-3’.

### Monitoring the general wellbeing, weight, and cardiac functions of the mice

Male and female wild type (+ / +), heterozygous (+/−), and homozygous (−/−) *Bag5* mutant mice were subject to monthly weight measurements. Echocardiography was performed to characterize cardiac functions of the mice when they reached three months of age, and this monitoring continued up to 12 months of age. In brief, mice were subjected to anesthesia via inhalation of isoflurane (1.5% in oxygen), the mouse chest was shaved and placed with ultrasound transmission gel. M-mode echocardiography was performed using Mindray Ultrasound and a 30-MHz transducer to access the following parameters: left ventricular fractional shortening (LVFS), left ventricular ejection fraction (LVEF), left ventricular internal diameter end diastole (LVIDd), left ventricular internal diameter end systole (LVIDs), end-diastolic volume (EDV), and end-systolic volume (ESV).

### Graded maximal exercise test (GXT)

GXT was performed to induce physical stress on the heart, modified from a previously reported protocol^14^. In brief, mice were acclimated to the treadmill for 5 days and rested for 2 days prior to performing GXT. On the test day, a shock grid was activated at 3 Hz and 1.5 mA, and mice were placed on the treadmill without speed for 3 min. A treadmill was then activated to a speed of 6 m/min for 5 min and increased to 9 m/min and 12 m/min for 2 min each. Subsequently, mice were placed on a treadmill without speed and graded, with activated shock grid. Speeds and grades were then increased as follows: (0 m/min, 3 min, 0 degree), (6 m/min, 2 min, 0 degree), (9 m/min, 2 min, 5 degree), (12 m/min, 2 min, 10 degree), (15 m/min, 2 min, 15 degree), (18 m/min, 1 min, 15 degree), (21 m/min, 1 min, 15 degree), (24 m/min, 1 min, 15 degree), (27 m/min, 1 min, 15 degree), (30 m/min, 1 min, 15 degree), and (33 m/min, 1 min, 15 degree) respectively. When mice continuously contacted the shock grid for 5 s, they were considered exhausted and the GXT was stopped.

### Induction of ER stress in mice using tunicamycin

Tunicamycin Ready Made Solution (5 mg/mL in DMSO (Sigma-Aldrich, MO, USA)) was diluted with 150 mM dextrose for injection. Three male and female homozygous *Bag5* KI (*Bag5−/−*)*,* heterozygous (*Bag5* +/−), and wild-type (WT, *Bag5* + */* +) mice at 12 weeks old were injected intraperitoneally with 2 mg/kg body weight of tunicamycin or the equivalent volume of vehicle (150 mM Dextrose)^22^.

Cardiac functions were determined using electrocardiogram (ECG) and echocardiography, which were performed before and after 72 h of tunicamycin (TN) injection (2 mg/kg body weight). Mice were anesthetized with inhalation of isoflurane (1.5% in oxygen) and placed in a supine position. A non-invasive ECG was performed at a speed of 50 mm/s. The needle electrodes were inserted subcutaneously into four limbs, and ECG was recorded using CardiMax FX-7102 (Fukuda Denshi Co. Ltd., Tokyo, Japan). Following a brief resting period of 2–3 min, echocardiography was carried out.

### Histological analysis and transmission *electron* microscopy (TEM)

Three hearts of each of the 12 groups of mice (three genotypes: +*/*+, + /− and *−/−*; two sexes: male and female; and two groups of intervention: TN and sham), totaling 36 hearts were studied. A portion of each heart was fixed with 10% neutral buffered formalin, paraffin-embedded, sectioned at 3 µm, and stained with hematoxylin and eosin (H&E). Slides were visualized under a light microscopy. In addition, heart tissues were fixed with 3% glutaraldehyde and processed following the standard protocol. Subsequently, they were examined using TEM (JEOL JEM-1400Plus, MA, USA).

### Western blot analysis

One heart of each of the 12 groups of mice (three genotypes: +*/*+, + /- and *−/−*; two sexes: male and female; and two groups of intervention: TN and sham), totaling 12 hearts was homogenized with a hand homogenizer grinder in RIPA lysis buffer (Thermo Scientific, MA, USA) containing a protease inhibitor and phosphatase inhibitor cocktail (Cell Signaling Technology, MA, USA), and centrifuged at 14,000 rpm for 10 min at 4 °C. Total protein concentration was determined by the bicinchoninic acid (BCA) assay (Thermo Scientific). Equal amounts of extracted protein (30 ug) was loaded on 10% SDS–polyacrylamide gel electrophoresis (SDS-PAGE), then transferred to polyvinylidene fluoride (PVDF) membranes using the iBlot 2 Dry Blotting System (Thermo Scientific). The membranes were blocked with 5% non-fat dry milk or 5% bovine serum albumin (BSA) and incubated with the following primary antibodies: BAG5 (#H00009529-D01P, Abnova, USA), GRP78 (#3177, Cell Signaling, USA), CHOP (#2895, Cell Signaling, USA), and GAPDH (#5174, Cell Signaling, USA) in 1:1000 dilution as a loading control. After being washed with TBST, the membranes were probed with horseradish peroxidase conjugated anti-rabbit (#7074, Cell Signaling, USA) or anti-mouse (#7076, Cell Signaling, USA) secondary antibody in 1:2000 dilution, and visualized with enhanced chemiluminescence reagents (Thermo Scientific) using the ImageQuant Las 4000 chemi-image (GE Healthcare).

### Apoptosis

The terminal deoxynucleotidyl transferase dUTP nick end labeling (TUNEL) assay was performed with the In Situ Cell Death Detection Kit, Fluorescein (Roche, Basel, Switzerland) on paraffin-embedded sections of TN-treated heart samples from six mice (one heart from each sex and each of the three genotypes). Sections were dewaxed, dehydrated in graded alcohol, and pretreated with proteinase K working solution (20 ug/ml in 10 mM Tris/HCL pH 8.0) for 15 min at room temperature. Sections were rinsed with Phosphate-buffered saline (PBS) for 3 times and incubated in 50 uL TUNEL reaction mixture for 60 min at 37 °C. After being rinsed with PBS for 3 times, sections were counterstained with Hoechst 33342 (H3570, Life Technologies, Eugene, OR, USA) (1:2000, in water) to visualize nuclei for 15 min at room temperature, rinsed with PBS for 3 times and then were mounted with the ProLong Gold antifade reagent (Invitrogen). Fluorescence imaging was performed by a confocal laser scanning microscope (Zeiss LSM800, Carl Zeiss, Germany) equipped with a 63 × oil immersion lens. Images were acquired using the Zen 3.4 (blue edition) software. Percentage of apoptosis was determined by counting TUNEL-positive cells per total cells in 5 fields per section (2 sections per heart sample) at 40 × magnification.

### Statistical analysis

Data was presented as mean ± standard deviation (SD). An independent sample t test was used to compare two independent means. Paired t-test was utilized to compare the pre- and post-tunicamycin injection data within each group. For multiple group comparisons, one-way analysis of variance (ANOVA), followed by Fisher’s least significant difference (LSD) test. Statistical significance was determined using SPSS. Differences were considered significant as follows: *P < 0.05; **P < 0.01; and ***P < 0.001, n.s., not significant.

### Supplementary Information

Supplementary Information.

## Data Availability

Data available within the article or its supplementary materials.

## References

[1] Hershberger RE, Hedges DJ, Morales A (2013). Dilated cardiomyopathy: The complexity of a diverse genetic architecture. Nat. Rev. Cardiol..

[2] Stroeks S (2023). Prevalence and clinical consequences of multiple pathogenic variants in dilated cardiomyopathy. Circ. Genom. Precis. Med..

[3] Hakui H (2022). Loss-of-function mutations in the co-chaperone protein BAG5 cause dilated cardiomyopathy requiring heart transplantation. Sci. Transl. Med..

[4] Gupta MK (2016). GRP78 interacting partner Bag5 responds to ER stress and protects cardiomyocytes from ER stress-induced apoptosis. J. Cell Biochem..

[5] Knezevic T (2015). BAG3: A new player in the heart failure paradigm. Heart Fail. Rev..

[6] Townsend PA, Cutress RI, Sharp A, Brimmell M, Packham G (2003). BAG-1 prevents stress-induced long-term growth inhibition in breast cancer cells via a chaperone-dependent pathway. Cancer Res.

[7] Hakui H (2022). Refractory ventricular arrhythmias in a patient with dilated cardiomyopathy caused by a nonsense mutation in BAG5. Circ. J..

[8] Inoue S (2023). Compound heterozygous truncating variants in the BAG5 gene as a cause of early-onset dilated cardiomyopathy. Circ. Genom. Precis. Med..

[9] Wang S (2018). Endoplasmic reticulum stress in the heart: Insights into mechanisms and drug targets. Br. J. Pharmacol..

[10] Domínguez F (2018). Dilated cardiomyopathy due to BLC2-associated athanogene 3 (BAG3) mutations. J. Am. Coll. Cardiol..

[11] Czosek RJ (2016). Arrhythmic burden and ambulatory monitoring of pediatric patients with cardiomyopathy. Pacing Clin. Electrophysiol..

[12] Kumar S (2016). Long-term arrhythmic and nonarrhythmic outcomes of lamin A/C mutation carriers. J. Am. Coll. Cardiol..

[13] Gan S (2024). BAG5 regulates HSPA8-mediated protein folding required for sperm head-tail coupling apparatus assembly. EMBO Rep..

[14] Petrosino JM (2016). Graded maximal exercise testing to assess mouse cardio-metabolic phenotypes. PLoS One.

[15] Hamada H (2004). Dilated cardiomyopathy caused by aberrant endoplasmic reticulum quality control in mutant KDEL receptor transgenic mice. Mol. Cell Biol..

[16] Fu HY (2010). Ablation of C/EBP homologous protein attenuates endoplasmic reticulum-mediated apoptosis and cardiac dysfunction induced by pressure overload. Circulation.

[17] Oyadomari S, Mori M (2004). Roles of CHOP/GADD153 in endoplasmic reticulum stress. Cell Death Differ..

[18] D'Oria R (2020). The role of oxidative stress in cardiac disease: From physiological response to injury factor. Oxid. Med. Cell Longev..

[19] Kanchanasevee C (2020). Phenotypic and genotypic features of thai patients with nonsyndromic tooth agenesis and WNT10A variants. Front. Physiol..

[20] Nitayavardhana I (2020). Four novel mutations of FAM20A in amelogenesis imperfecta type IG and review of literature for its genotype and phenotype spectra. Mol. Genet. Genomics.

[21] Verdonschot JAJ (2020). Implications of genetic testing in dilated cardiomyopathy. Circ. Genom. Precis. Med..

[22] Prola A (2019). Endoplasmic reticulum stress induces cardiac dysfunction through architectural modifications and alteration of mitochondrial function in cardiomyocytes. Cardiovasc. Res..

