# Supplementary material for: A novel BAG5 variant impairs the ER stress response pathway, causing dilated cardiomyopathy and arrhythmia
Source: Sci Rep. 2024 May 25;14:11980. doi: 10.1038/s41598-024-62764-y (PMC11127938; doi:10.1038/s41598-024-62764-y)
Supplement: Supplementary file 1 — Supplementary Information. [file 41598_2024_62764_MOESM1_ESM.pdf]

SUPPLEMENTAL MATERIAL

A Novel BAG5 Variant Impairs the ER Stress Response Pathway, Causing Dilated Cardiomyopathy and Arrhythmia

Rutairat Wongong[1,2]; Anusak Kijtawornrat[3]; Chalurmpon Srichomthong[2]; Siraprapa Tongkobpeth[1]; Phichittra Od-Ek[2]; Adjima Assawapitaksakul[2]; Natarin Caengprasath[1,2]; Apichai Khongphatthanayothin[4]; Thantrira Porntaveetus[5,*]; and Vorasuk Shotelersuk[1,2]

[1]Center of Excellence for Medical Genomics, Department of Pediatrics, Faculty of Medicine, Chulalongkorn University, Bangkok, Thailand

[2]Excellence Center for Genomics and Precision Medicine, King Chulalongkorn Memorial Hospital, the Thai Red Cross Society, Bangkok, Thailand

[3]Department of Physiology, Faculty of Veterinary Science, Chulalongkorn University, Bangkok, Thailand

[4]Center of Excellence in Arrhythmia Research, Department of Pediatrics, Faculty of Medicine, Chulalongkorn University, Bangkok, Thailand

[5]Center of Excellence in Genomics and Precision Dentistry, Department of Physiology, Faculty of Dentistry, Chulalongkorn University, Bangkok, 10330, Thailand.

SUPPLEMENTAL FIGURES

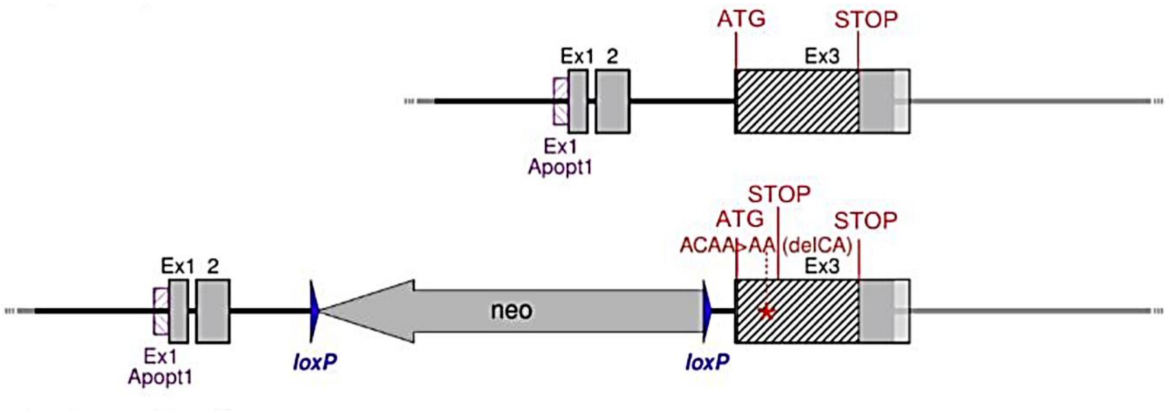

Figure S1: Schematic representation of the selected targeting strategy for the generation of *Bag5* mutant knock-in mice. The diagram is not depicted to scale. Hatched rectangles: Bag5 coding sequences, Grey rectangles: Non-coding exon portions, Solid lines: Chromosomal sequences, Pink rectangles: Exons of neighboring genes, Blue triangles: LoxP sites, Red asterisk: the delCA deletion. Initiation (ATG) codon, stop (STOP) codons, and neomycin (neo) positive selection cassette are indicated.

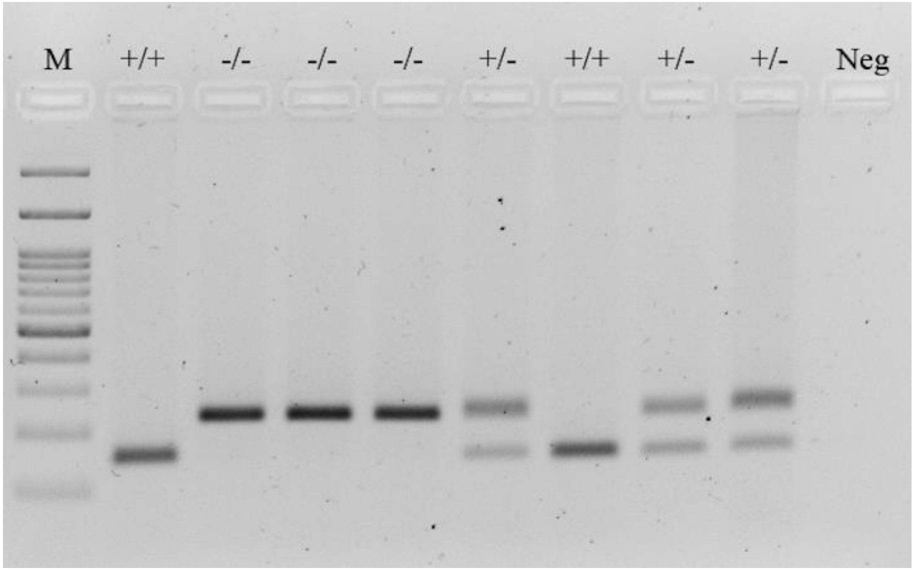

Figure S2: Genotyping. The polymerase chain reaction (PCR) genotyping was performed on the offspring at 3 weeks of age to differentiate *Bag5+/+*, *Bag5+/-*, and *Bag5-/-* mice. Ear or tail tissues were extracted and amplified using KAPA Mouse Genotyping Kit (KAPA Biosystems, #KK7302) with genotyping primers as follows: 5'-GGTGCCTCTGAAG ACAGAAAGGGAATC-3' and 5'-AAGCTGGGGCGGAGCTGGAGA-3'. The estimated length of *Bag5+/+* is 152 bp (lower) and the *Bag5-/-* is 232 bp (upper). M = marker, Neg = negative control.

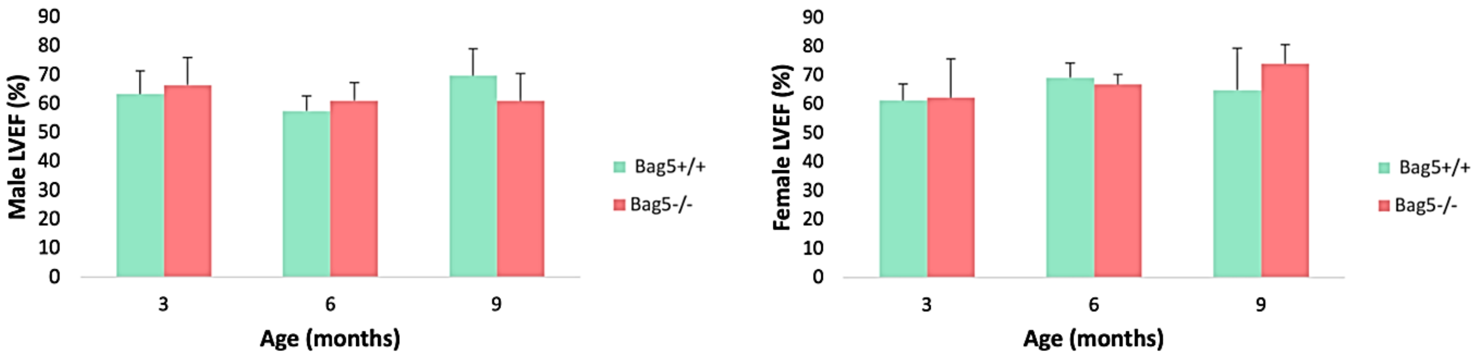

Figure S3: Echocardiographic assessment of baseline mouse cardiac function. Left ventricular ejection fraction (LVEF) in male and female of *Bag5+/+*, *Bag5+/-*, and *Bag5-/-* mice at 3, 6 and 9 months of age. Results are shown as mean ± SD. At 3 months of age, n = 6 for male, n = 4 for female. At 6 months of age, n = 2 for male, n = 4 for female. At 9 months of age, n = 2 for male, n = 3 for female. Statistical analysis was determined using independent sample t test.

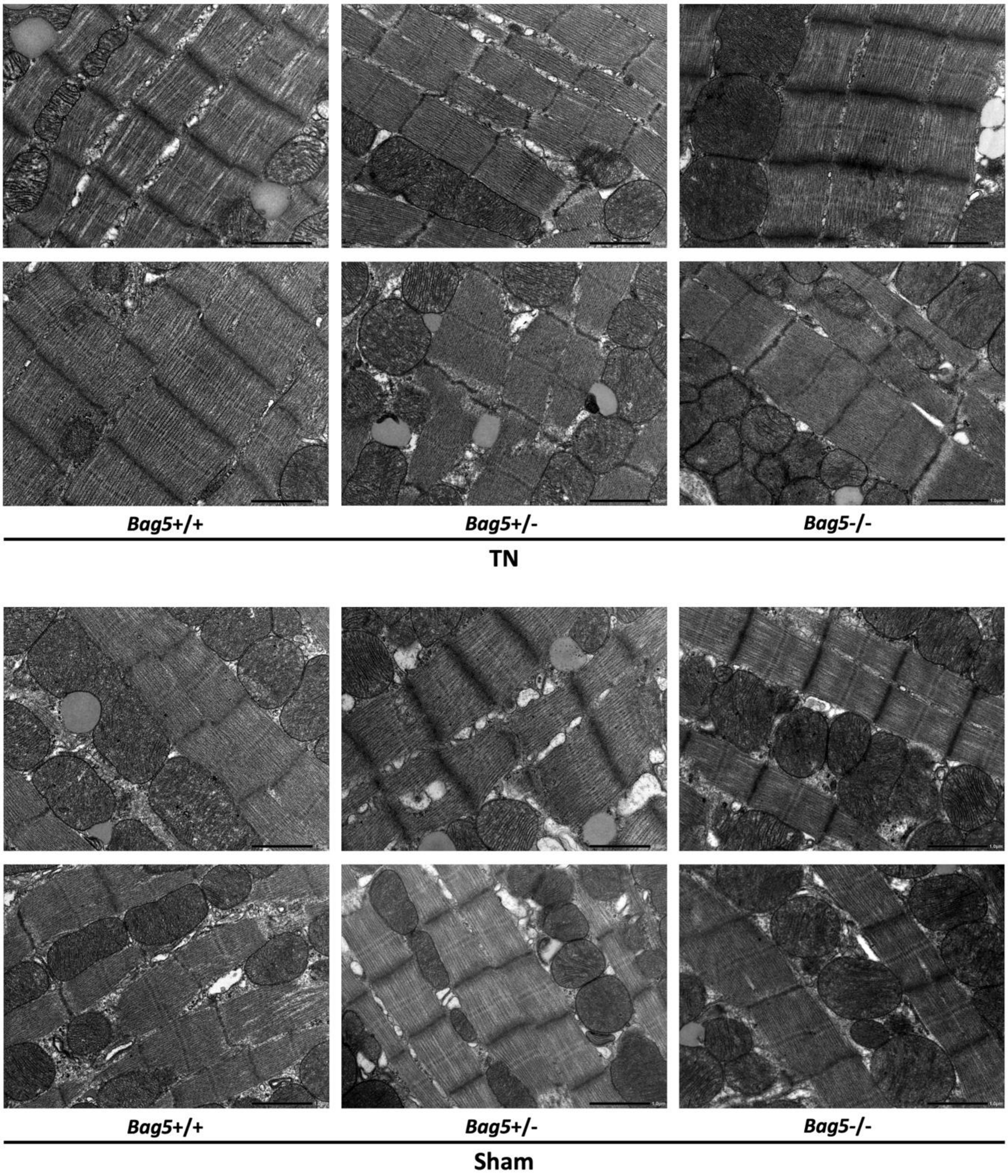

Figure S4: Histological analysis of mouse hearts. Representative transmission electron microscopy (TEM) images of cardiac tissue sections in male (upper) and female (lower) mice from *Bag5+/+, Bag5+/-,* and *Bag5-/-* groups following tunicamycin (TN) injection and without TN injection (sham controls), all at 3 months of age. Scale bars, 1 μm.

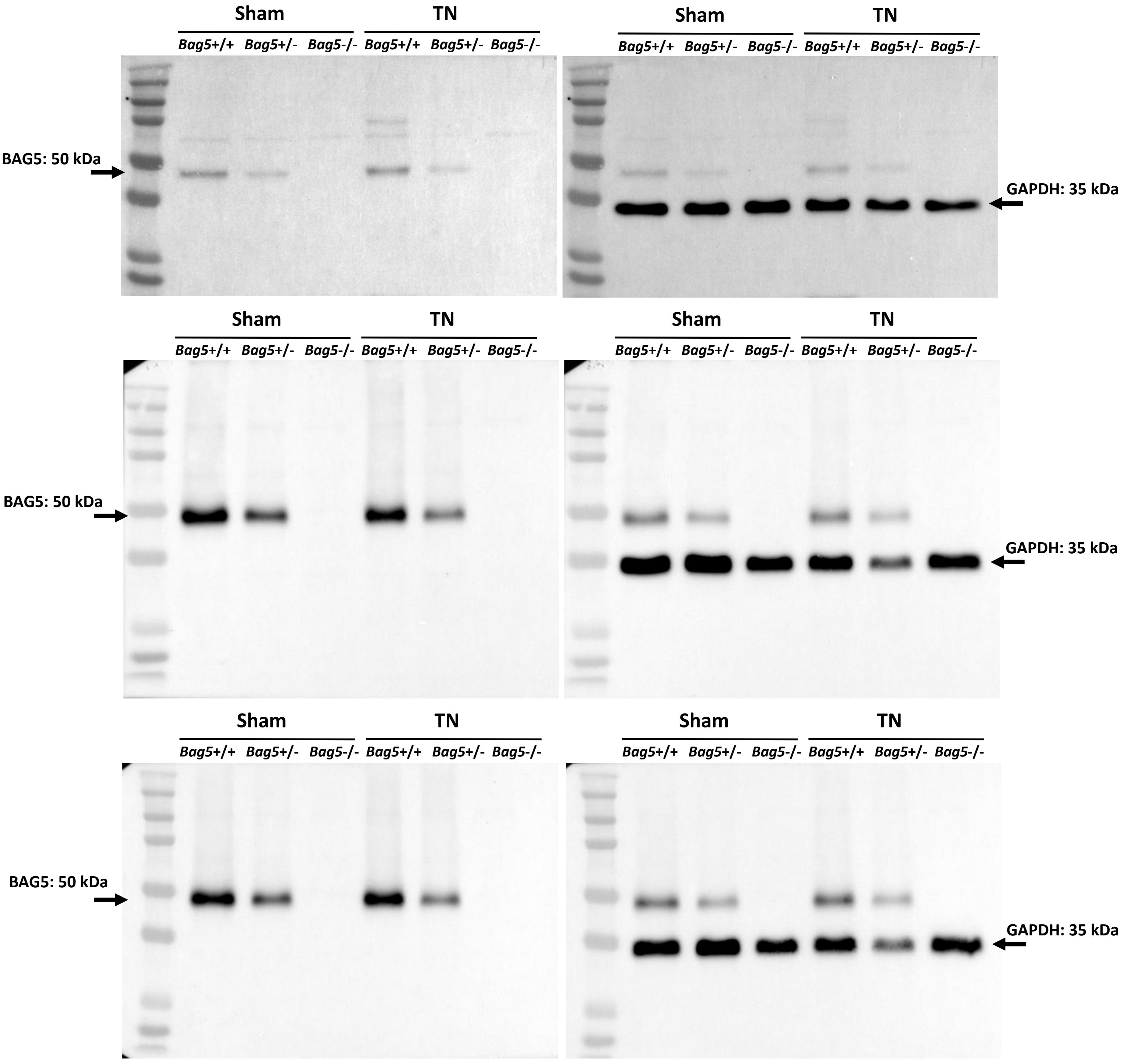

Figure S5: Western blotting. BAG5/GAPDH in male mice.

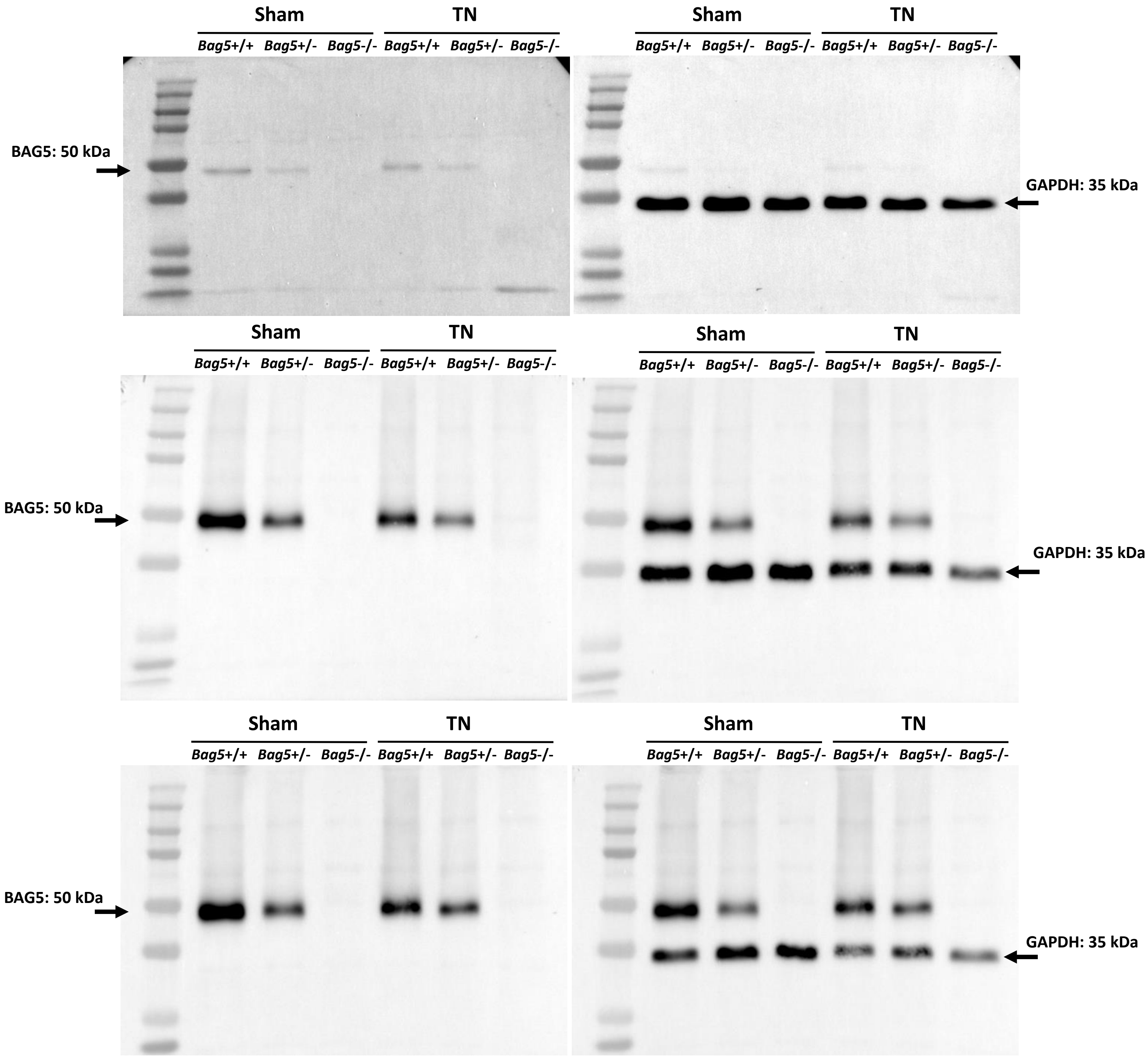

Figure S5 (Continued 1): Western blotting. BAG5/GAPDH in female mice.

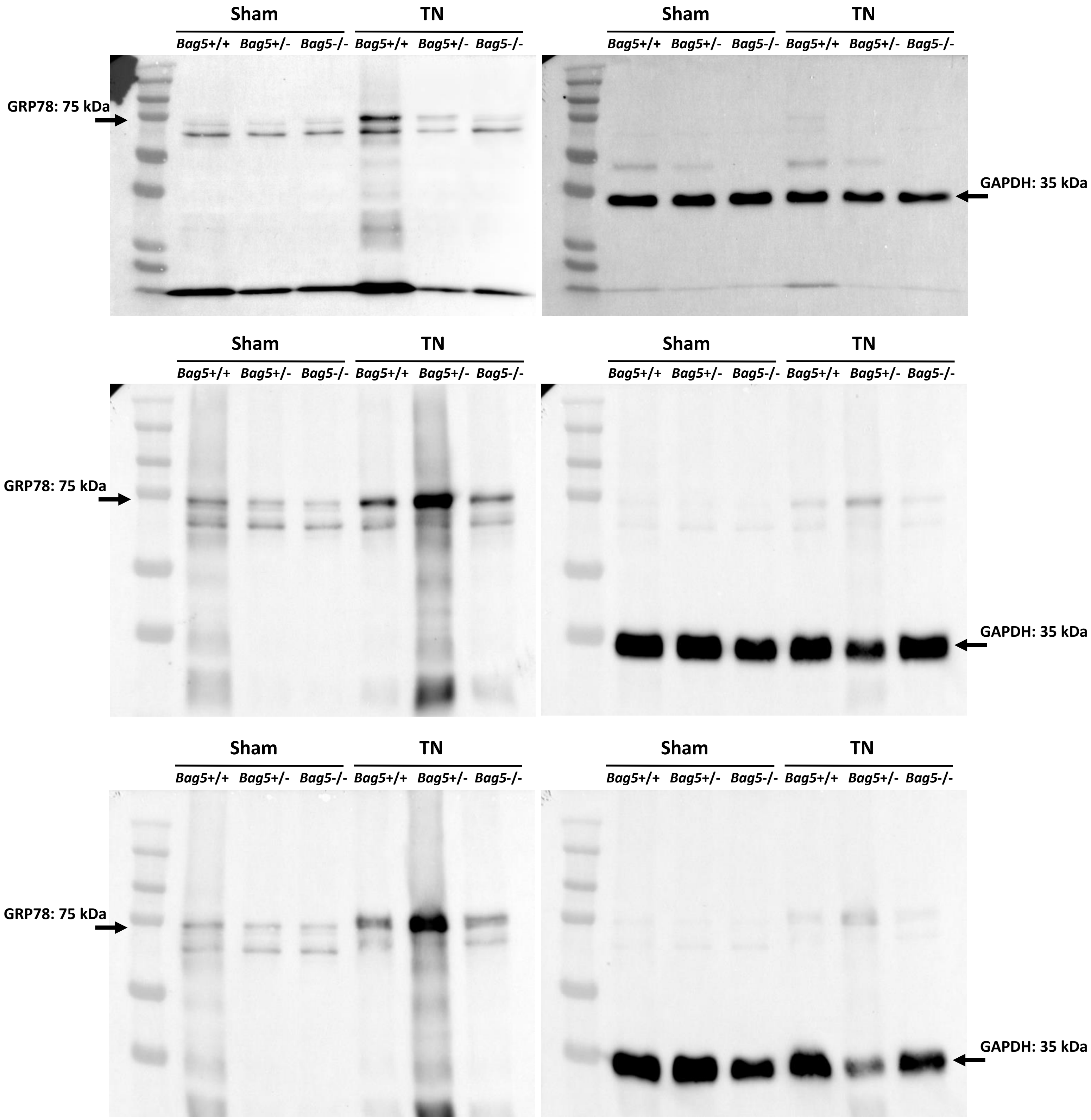

Figure S5 (Continued 2): Western blotting. GRP78/G in male mice.

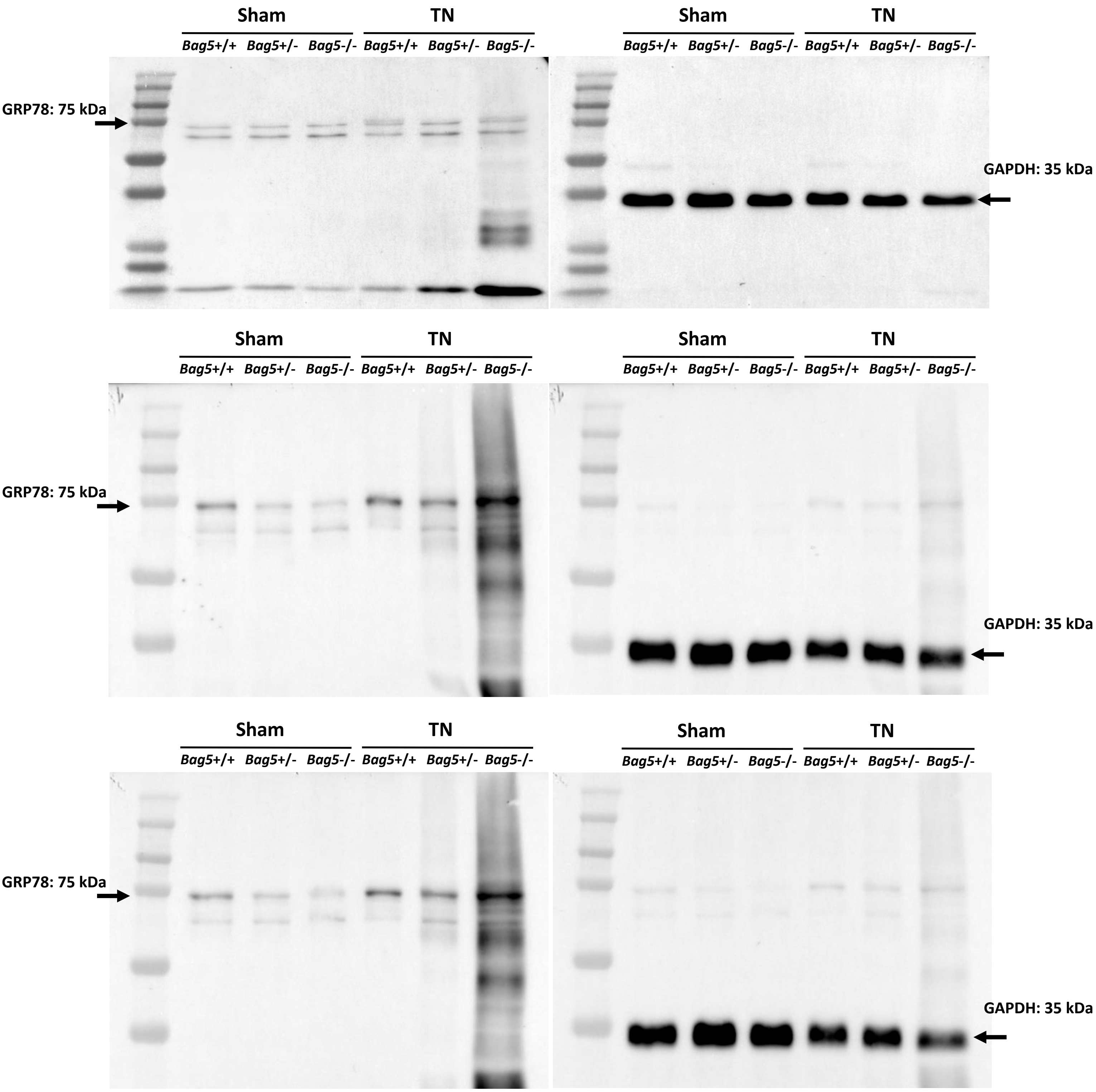

Figure S5 (Continued 3): Western blotting. GRP78/GAPDH in female mice.

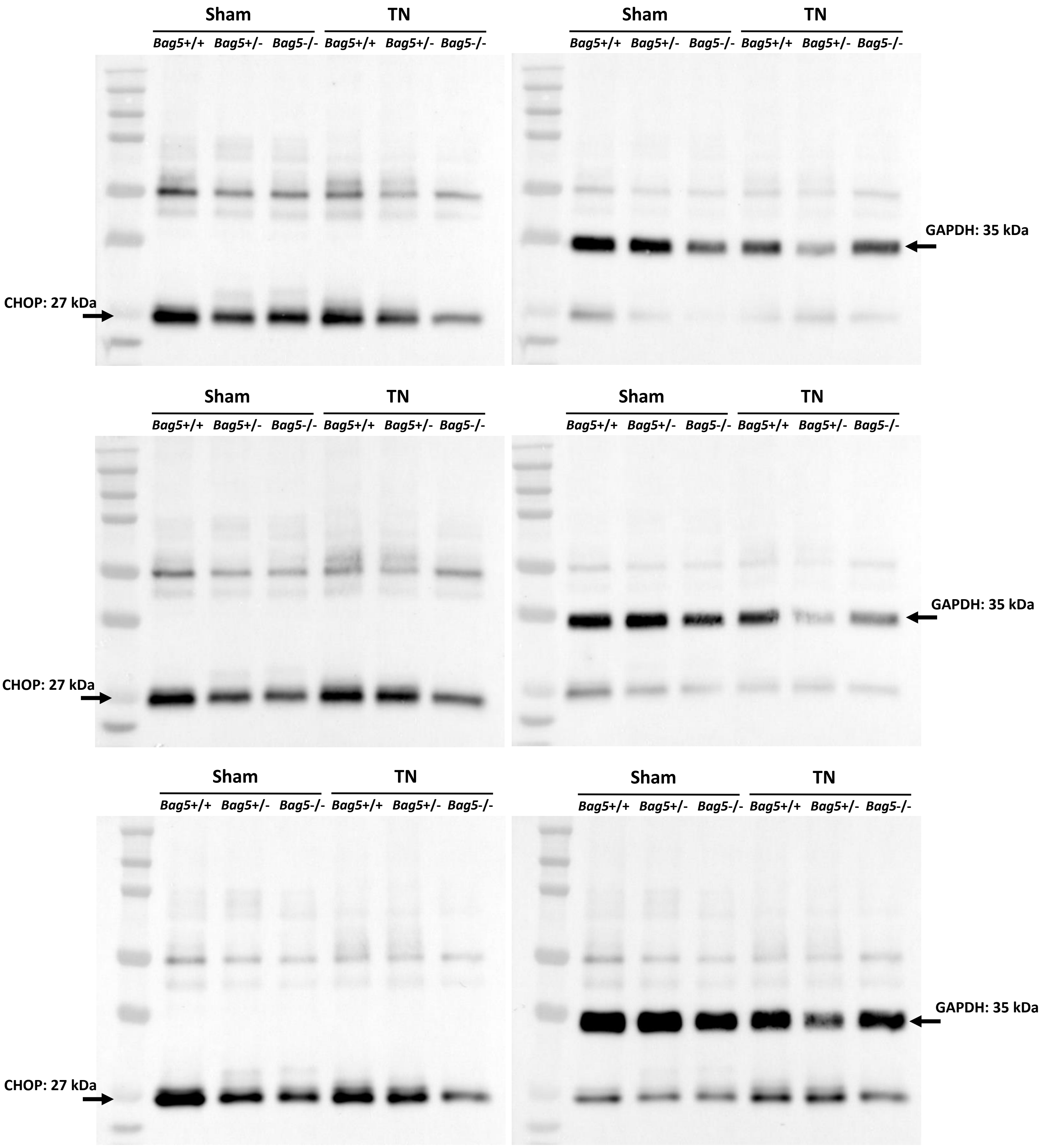

Figure S5 (Continued 4): Western blotting. CHOP/GAPDH in male mice.

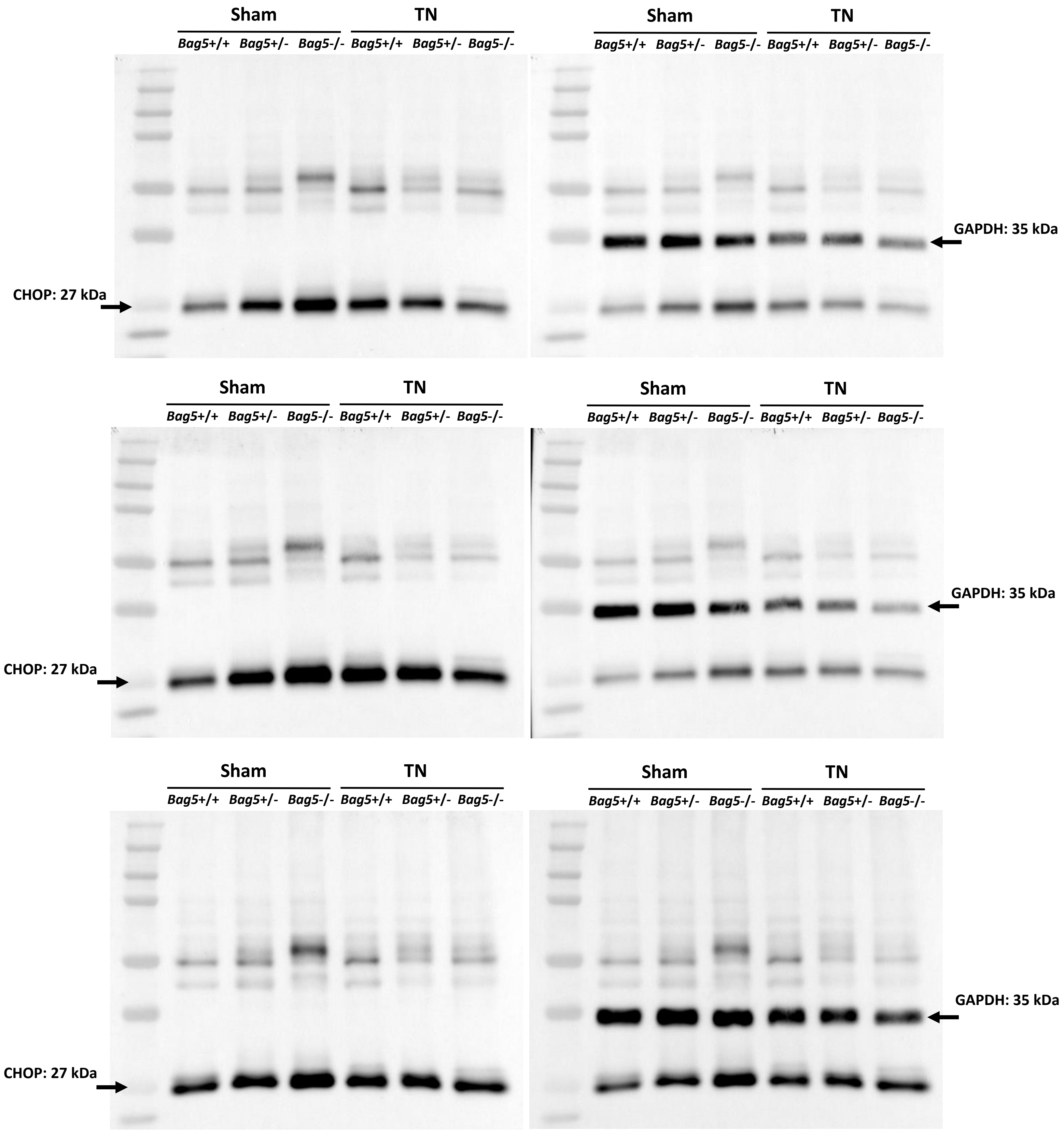

Figure S5 (Continued 5):): Western blotting. CHOP/GAPDH in female mic

SUPPLEMENTAL TABLES

Table S1: Details of participants in the study

DCM Family	Gender	Age (years)	Zygosity	LVEF (%)	LVFS (%)	EDV (ml)	ECG
**Father (II-13)**	Male	39	Heterozygous	64.79	35.51	112.47	ECG without pathological findings
**Mother (II-14)**	Female	37	Heterozygous	76.37	44.56	70.77	Non-specific T wave abnormality
**Affected (III-2)**	Female	20	Homozygous mutant	46.00	32.02	94.66	Low Voltage in Limb Leads, ST Lowering in lateral Leads
**Proband (III-4)**	Male	17	Homozygous mutant	40.00	19.00	143.00	Low Voltage in Limb Leads, anteroseptal Infarct, inferior Infarct in Stage 3
**Proband (III-5)**	Male	15	Homozygous mutant	35.75	17.31	142.18	Low Voltage in Limb Leads, anteroseptal Infarct in Stage 3
**Unaffected (III-7)**	Female	13	Heterozygous	59.00	31.00	75.10	ECG without pathological findings
**Unaffected (III-8)**	Female	12	Homozygous wildtype	61.00	31.00	65.50	ECG without pathological findings
**Unaffected (III-9)**	Female	9	Heterozygous	66.00	36.00	58.50	Inverted T wave in V1-V4
**Unaffected (III-12)**	Male	2	Heterozygous	69.00	38.00	35.30	Normal ECG

ECG: electrocardiogram, DCM: dilated cardiomyopathy, EDV: end-diastolic volume, LVEF: left ventricular ejection fraction, LVFS: left ventricular fractional shortening, ml: milliliter.

Table S2: Exercise end points parameters (± SD) from the graded maximal exercise test (GXT)

**Male (n=23)**	**Max run speed (m/m)**	**Distance (m)**	**Body weight (g)**
Bag5+/+ (n=7)	31.93 ± 2.23	24.07 ± 11.15	44.70 ± 5.53
Bag5+/- (n=10)	33.00 ± 0.00	30.45 ± 4.68	40.58 ± 5.75
Bag5-/- (n=6)	33.00 ± 0.00	31.83 ± 1.99	40.24 ± 6.52

**Female (n=29)**	**Max run speed (m/m)**	**Distance (m)**	**Body weight (g)**
Bag5+/+ (n=8)	33.00 ± 0.00	31.69 ± 2.65	32.24 ± 6.06
Bag5+/- (n=17)	33.00 ± 0.00	31.74 ± 2.05	31.34 ± 3.43
Bag5-/- (n=4)	33.00 ± 0.00	31.50 ± 3.16	36.27 ± 4.46

SD: standard deviation, m/m: meter/minute, m: meter, g: gram.

Table S3: Echocardiographic measurements before and after tunicamycin (TN) injection in male mice.

Male	*Bag5+/+* (n=3)			*Bag5+/-* (n=3)			*Bag5-/-* (n=3)		
	**Before TN**	**After TN**	***p*-value**	**Before TN**	**After TN**	***p*-value**	**Before TN**	**After TN**	***p*-value**
LVEF (%)	75.57 ± 0.66	69.41 ± 0.42†††	<0.001	69.64 ± 0.44	65.79 ± 1.09†	0.033	60.34 ± 0.21*** ###	49.27 ± 3.67*** ###	0.161
LVFS (%)	38.44 ± 0.56	33.52 ± 0.24†	0.002	33.76 ± 0.38	30.80 ± 0.81†	0.038	27.38 ± 1.59*** ###	21.05 ± 2.27*** ###	0.176
LVIDd (mm)	3.00 ± 0.20	2.83 ± 0.38	0.370	3.27 ± 0.29	2.63 ± 0.21	0.156	3.27 ± 0.00	2.9 ± 0.71	0.356
LVIDs (mm)	1.83 ± 0.15	1.90 ± 0.26	0.635	2.20 ± 0.17	1.83 ± 0.12	0.159	2.37 ± 0.00	2.30 ± 0.57	0.500
EDV (ml)	0.07 ± 0.01	0.06 ± 0.02	0.529	0.09 ± 0.02	0.05 ± 0.01	0.145	0.1 ± 0.05	0.08 ± 0.05	0.430
ESV (ml)	0.02 ± 0.01	0.02 ± 0.01	N/A	0.03 ± 0.01	0.02 ± 0.01	0.423	0.04 ± 0.02	0.04 ± 0.02	0.500
HR (bpm)	563 ± 22.11	476 ± 39.59	0.131	545 ± 11.37	416 ± 57.74	0.084	505 ± 74.47	386 ± 45.96	0.131

Values are mean ± SD. *p*-values were calculated using one-way ANOVA and paired t-test.

***$p < 0.001$, *$p < 0.05$: *Bag5-/-* vs *Bag5+/+* under the same condition.

###$p < 0.001$, #$p < 0.05$: *Bag5-/-* vs *Bag5+/-* under the same condition.

†††$p < 0.001$, †$p < 0.05$: post-TN vs pre-TN.

LVEF: left ventricular ejection fraction, LVFS: left ventricular fractional shortening, LVIDd: left ventricle internal diameter at diastole, LVIDs: left ventricle internal diameter at systole, EDV: end-diastolic volume, ESV: end-systolic volume, HR: heart rate, TN: tunicamycin, SD: standard deviation, N/A: not available. N = 3 per group, except male *Bag5-/-* (after TN), where N = 2 due to one animal death post-TN injection.

Table S4: Echocardiographic measurements before and after tunicamycin (TN) injection in female mice.

Female	***Bag5+/+*** **(n=3)**			***Bag5+/-*** **(n=3)**			***Bag5-/-*** **(n=3)**		
	**Before TN**	**After TN**	***P*-value**	**Before TN**	**After TN**	***P*-value**	**Before TN**	**After TN**	***P*-value**
LVEF (%)	75.74 ± 0.97	67.84 ± 1.31†	0.012	69.36 ± 1.90	68.61 ± 0.85	0.358	60.45 ± 1.53*** ###	50.86 ± 2.26*** ###†	0.005
LVFS (%)	38.50 ± 0.87	32.11 ± 0.99†	0.012	33.45 ± 1.46	32.73 ± 0.50	0.330	27.22 ± 1.01*** ###	21.72 ± 1.28*** ###†	0.004
LVIDd (mm)	2.77 ± 0.15	2.17 ± 0.21†	0.035	2.90 ± 0.26	2.40 ± 0.36	0.185	2.40 ± 0.26	2.90 ± 0.26†	0.038
LVIDs (mm)	1.70 ± 0.10	1.47 ± 0.12	0.118	1.93 ± 0.15	1.60 ± 0.26	0.199	1.73 ± 0.15	2.27 ± 0.15†	0.004
EDV (ml)	0.06 ± 0.01	0.03 ± 0.01†	0.015	0.06 ± 0.02	0.04 ± 0.02	0.184	0.04 ± 0.01#	0.07 ± 0.02*#†	0.035
ESV (ml)	0.01 ± 0.00	0.01 ± 0.00	N/A	0.02 ± 0.00	0.01 ± 0.01	0.184	0.01 ± 0.01#	0.03 ± 0.01*#†	0.038
HR (bpm)	521 ± 94.32	495 ± 120.79	0.794	517 ± 21.94	436 ± 33.65	0.097	418 ± 50.46	424 ± 61.07	0.615

Values are mean ± SD. *p*-values were calculated using one-way ANOVA and paired t-test.

***$p < 0.001$, *$p < 0.05$: *Bag5-/-* vs *Bag5+/+* under the same condition.

###$p < 0.001$, #$p < 0.05$: *Bag5-/-* vs *Bag5+/-* under the same condition.

†††$p < 0.001$, †$p < 0.05$: post-TN vs pre-TN

LVEF: left ventricular ejection fraction, LVFS: left ventricular fractional shortening, LVIDd: left ventricle internal diameter at diastole, LVIDs: left ventricle internal diameter at systole, EDV: end-diastolic volume, ESV: end-systolic volume, HR: heart rate, TN: tunicamycin, SD: standard deviation, N/A: not available.

Table S5: Number of humans and mice with abnormal ECG findings across different genotypes.

**Gender and Genotypes**	**Abnormal ECG in humans**	**Abnormal ECG in mice (following TN injection)**
Male +/-	0/2	**1/3**
Female +/-	**2/3**	0/3
Male -/-	2/2	1/3
Female -/-	1/1	0/3

ECG: electrocardiogram, TN: tunicamycin
